# A Lipid Emulsion Reverses Toxic-Dose Bupivacaine-Induced Vasodilation during Tyrosine Phosphorylation-Evoked Contraction in Isolated Rat Aortae

**DOI:** 10.3390/ijms18020394

**Published:** 2017-02-13

**Authors:** Seong-Ho Ok, Soo Hee Lee, Seong-Chun Kwon, Mun Hwan Choi, Il-Woo Shin, Sebin Kang, Miyeong Park, Jeong-Min Hong, Ju-Tae Sohn

**Affiliations:** 1 Department of Anesthesiology and Pain Medicine, Gyeongsang National University School of Medicine, Gyeongsang National University Hospital, 79 Gangnam-ro, Jinju 52727, Korea; mdoksh@naver.com (S.-H.O.); lishiuji@naver.com (S.H.L.); choibak@nate.com (M.H.C.); ilwooshin@hanmail.net (I.-W.S.); jebiny@naver.com (S.K.); 2 Department of Physiology, Institute of Clinical and Translational Research, Catholic Kwandong University, College of Medicine, Gangneung 25601, Korea; skwon2028@cku.ac.kr; 3 Department of Anesthesiology and Pain Medicine, Gyeongsang National University Changwon Hospital, Changwon 51472, Korea; ele93547@naver.com; 4 Department of Anesthesia and Pain Medicine, School of Medicine, Pusan National University, Biomedical Research Institute, Pusan National University Hospital, Busan 49241, Korea; ccarrot@hanmail.net; 5 Institute of Health Sciences, Gyeongsang National University, Jinju 52727, Korea

**Keywords:** lipid emulsion, bupivacaine, vasodilation, sodium orthovanadate, tyrosine kinase, myosin phosphatase target subunit, aorta

## Abstract

The goal of this in vitro study was to examine the effect of a lipid emulsion on toxic-dose bupivacaine-induced vasodilation in a model of tyrosine phosphatase inhibitor sodium orthovanadate-induced contraction in endothelium-denuded rat aortae and to elucidate the associated cellular mechanism. The effect of a lipid emulsion on vasodilation induced by a toxic dose of a local anesthetic during sodium orthovanadate-induced contraction was examined. In addition, the effects of various inhibitors, either bupivacaine alone or a lipid emulsion plus bupivacaine, on protein kinase phosphorylation induced by sodium orthovanadate in rat aortic vascular smooth muscle cells was examined. A lipid emulsion reversed the vasodilation induced by bupivacaine during sodium orthovanadate-induced contraction. The lipid emulsion attenuated the bupivacaine-mediated inhibition of the sodium orthovanadate-induced phosphorylation of protein tyrosine, c-Jun NH_2_-terminal kinase (JNK), myosin phosphatase target subunit 1 (MYPT1), phospholipase C (PLC) γ-1 and extracellular signal-regulated kinase (ERK). These results suggest that a lipid emulsion reverses toxic-dose bupivacaine-induced vasodilation during sodium orthovanadate-induced contraction via the activation of a pathway involving either tyrosine kinase, JNK, Rho-kinase and MYPT1 or tyrosine kinase, PLC γ-1 and ERK, and this reversal is associated with the lipid solubility of the local anesthetic and the induction of calcium sensitization.

## 1. Introduction

Lipid emulsions are widely used to mitigate systemic toxicity induced by local anesthetics or lipid-soluble non-local anesthetic drugs [1]. In agreement with the lipid sink theory, which is accepted as an underlying mechanism of lipid emulsion treatment, lipid emulsions reverse toxic-dose local anesthetic-induced vasodilation in a manner that is dependent on the lipid solubility of the local anesthetic [2,3].

Tyrosine kinase-induced protein tyrosine phosphorylation in vascular smooth muscle contributes to contraction by both calcium sensitization, which is mediated by the regulation of phospholipase C and D and mitogen-activated protein kinase (MAPK) activity, and the regulation of voltage-operated calcium channels [4]. Several contractile agonists, including norepinephrine, serotonin and angiotensin II, induce vasoconstriction mediated by protein tyrosine phosphorylation via the activation of non-receptor tyrosine kinases [5,6]. The tyrosine phosphatase inhibitor sodium orthovanadate causes tyrosine phosphorylation-mediated contraction via the inhibition of protein tyrosine dephosphorylation [6,7]. In addition, sodium orthovanadate-induced contraction involves Rho-kinase-induced myosin phosphatase target subunit 1 (MYPT1) phosphorylation, contributing to the inhibition of myosin light chain phosphatase, which is associated with calcium sensitization [8,9].

Vasodilation induced by anesthetics is mediated by either decreased calcium sensitization or reduced intracellular calcium concentrations [4]. However, the effect of lipid emulsions on toxic-dose bupivacaine-induced vasodilation during protein tyrosine phosphorylation-mediated contraction, which is associated with a mechanism of agonist-induced contraction, including norepinephrine released from sympathetic nerve endings in vivo, remains unknown [5,10]. Therefore, the goal of this in vitro study was to investigate the effect of lipid emulsion on vasodilation induced by a toxic dose of bupivacaine during sodium orthovanadate-induced protein tyrosine phosphorylation-mediated contraction of isolated endothelium-denuded rat aortae and to elucidate the associated cellular mechanism, with a particular focus on the signaling pathways downstream of sodium orthovanadate-induced protein tyrosine phosphorylation in vascular smooth muscle.

## 2. Results

**Figure 1:**
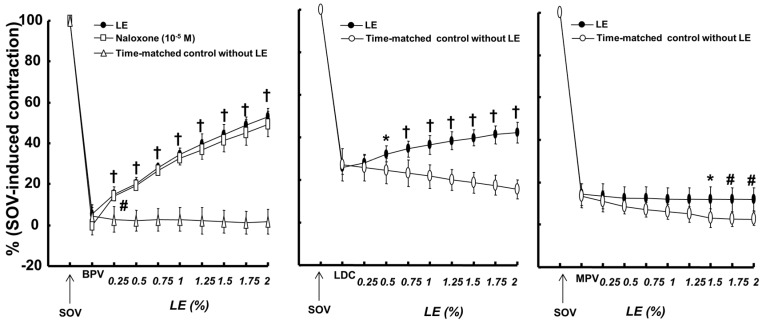
Effects of lipid emulsion (LE, 0.25% to 2%) on the vasodilation induced by a toxic dose of bupivacaine (BPV, 10^−3^ M, *N* = 5), lidocaine (LDC, 3 × 10^−3^ M, *N* = 6) and mepivacaine (MPV, 10^−2^ M, *N* = 6) during sodium orthovanadate (SOV, 10^−3^ M)-induced contraction in isolated endothelium-denuded rat aortae with or without naloxone. The data are expressed as the percentage of maximal SOV-induced contraction. The effects of the LE on the local anesthetic-induced vasodilation in SOV-induced contraction were analyzed using a two-way repeated-measures analysis of variance followed by Bonferroni’s post hoc test (Prism 5.0, GraphPad Software, San Diego, CA, USA). *N* indicates the number of rats from which the descending thoracic aortic rings were derived. * *p* < 0.05, # *p* < 0.01 and † *p* < 0.001 versus time-matched control without LE.

**Figure 2:**
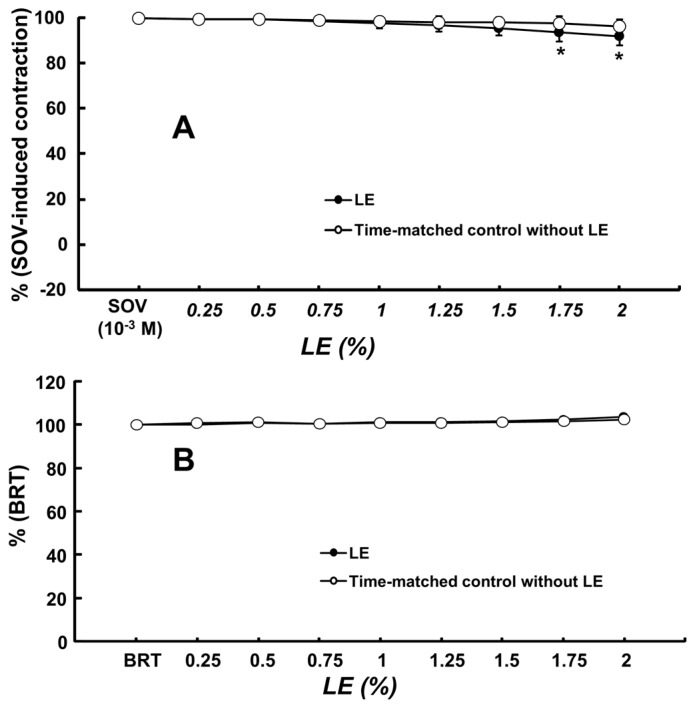
(**A**) Effect of lipid emulsion (LE, *N* = 8) alone on the contraction induced by sodium orthovanadate (SOV, 10^−3^ M) in isolated endothelium-denuded rat aortae. The data are shown as the means ± SD and are expressed as the percentage of maximal contraction induced by SOV. The effect of the LE alone on SOV-induced contraction or resting tension was analyzed using a two-way repeated-measures analysis of variance followed by Bonferroni’s post hoc test. *N* indicates the number of isolated endothelium-denuded rat aortae. * *p* < 0.01 versus time-matched control with LE; (**B**) Effect of LE (*N* = 6) alone on the baseline resting tension (BRT, 3.0 g) in isolated endothelium-denuded rat aortae without SOV. The data are shown as the means ± SD and are expressed as the percentage of BRT. *N* indicates the number of isolated endothelium-denuded rat aortae.

**Figure 3:**
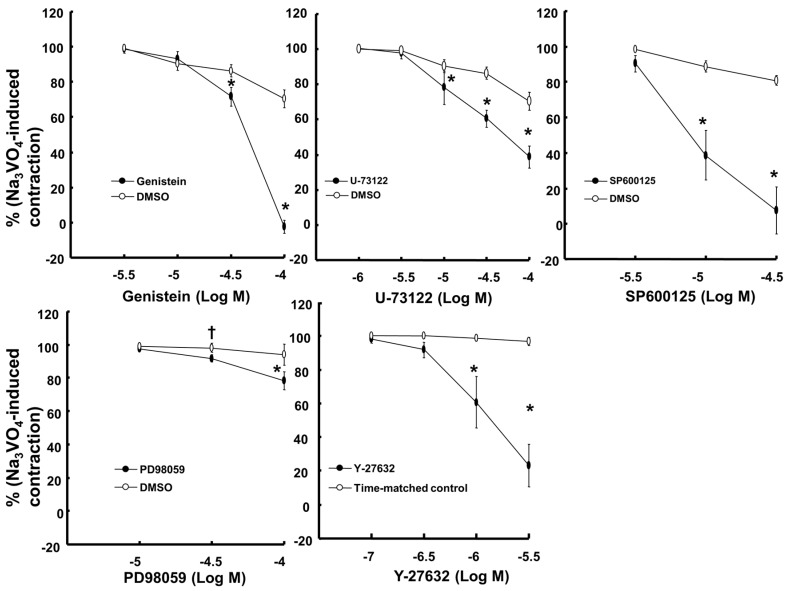
Effects of genistein (*N* = 6), U-73122 (*N* = 6), SP600125 (*N* = 6), PD98059 (*N* = 6) and Y-27632 (*N* = 6) on the contraction induced by sodium orthovanadate (Na_3_VO_4_, 10^−3^ M) in isolated endothelium-denuded rat aortae. The data are expressed as the percentage of Na_3_VO_4_-induced contraction. The effects of several inhibitors on Na_3_VO_4_-induced contraction were analyzed using a two-way repeated-measures analysis of variance followed by Bonferroni’s post hoc test. *N* indicates the number of descending thoracic aortic rings. † *p* < 0.01 and * *p* < 0.001 versus dimethyl sulfoxide (DMSO) or a time-matched control.

Bupivacaine (10^−3^ M), lidocaine (3 × 10^−3^ M) and mepivacaine (10^−2^ M) produced vasodilation in isolated endothelium-denuded rat aortae precontracted with sodium orthovanadate (10^−3^ M) (Figure 1). The lipid emulsion reversed bupivacaine- and lidocaine-induced vasodilation during sodium orthovanadate-induced vasoconstriction (Figure 1; bupivacaine: *p* < 0.001 versus time-matched control without lipid emulsion at 0.25% to 2% lipid emulsion; lidocaine: *p* < 0.05 versus time-matched control without lipid emulsion at 0.5% to 2% lipid emulsion), whereas a high concentration (1.5% to 2%) of the lipid emulsion alone slightly reversed mepivacaine-induced vasodilation (Figure 1; *p* < 0.05). In addition, naloxone (10^−5^ M) had no effect on the lipid emulsion-mediated reversal of bupivacaine-induced vasodilation (Figure 1). The magnitude of the lipid emulsion (0.5% to 2%)-mediated reversal of vasodilation induced by local anesthetics was as follows: bupivacaine, lidocaine and mepivacaine (Figure S1). Lipid emulsion (1.75% and 2%) alone slightly decreased sodium orthovanadate-induced contraction (*p* < 0.01; Figure 2A). However, lipid emulsion itself had no effect on baseline resting tension (Figure 2B). The tyrosine kinase inhibitor genistein (3 × 10^−5^ and 10^−4^ M), the c-Jun NH_2_-terminal kinase (JNK) inhibitor SP600125 (10^−5^ and 3 × 10^−5^ M) and the Rho-kinase inhibitor Y-27632 (10^−6^ and 3 × 10^−6^ M) greatly inhibited sodium orthovanadate (10^−3^ M)-induced contraction (Figure 3; *p* < 0.001 versus dimethyl sulfoxide (DMSO) or time-matched control). In addition, the phospholipase C (PLC) γ-1 inhibitor U-73122 (10^−5^ to 10^−4^ M) and the extracellular signal-regulated kinase (ERK) inhibitor PD98059 (3 × 10^−5^ and 10^−4^ M) attenuated sodium orthovanadate (10^−3^ M)-induced contraction (Figure 3; *p* < 0.01 versus DMSO).

**Figure 4:**
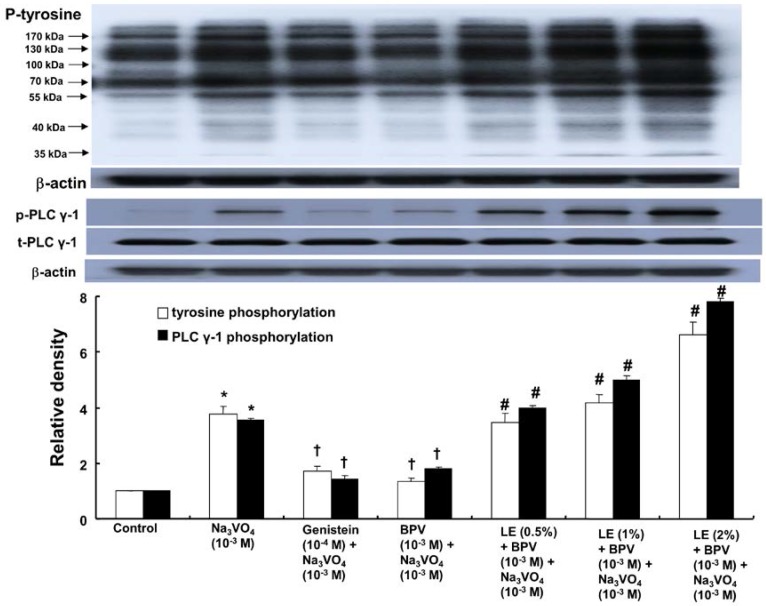
Effects of genistein alone, bupivacaine (BPV) alone and combined treatment with lipid emulsion (LE) and BPV on protein tyrosine and phospholipase C (PLC) γ-1 phosphorylation induced by sodium orthovanadate (Na_3_VO_4_, 10^−3^ M) in rat aortic vascular smooth muscle cells (RAVSMCs). RAVSMCs were treated with Na_3_VO_4_ alone for 30 min, pretreated with genistein or BPV for 1 h followed by treatment with Na_3_VO_4_ for 30 min or pretreatment with LE (0.5%, 1% and 2%) for 1 h followed by BPV for 1 h and subsequent treatment with Na_3_VO_4_ for 30 min. Protein tyrosine and PLC γ-1 (Tyr783) phosphorylation was examined by Western blot analysis as described in the Materials and Methods. The data are expressed as the means ± SD (*N* = 3). The effects of genistein, BPV and combined treatment with LE and BPV on Na_3_VO_4_-induced protein tyrosine and PLC γ-1 phosphorylation were analyzed using a one-way analysis of variance followed by Bonferroni’s post hoc test. *N* indicates the number of independent experiments. * *p* < 0.001 versus control. † *p* < 0.001 versus Na_3_VO_4_. # *p* < 0.001 versus combined treatment with BPV and Na_3_VO_4_. p-tyrosine: phosphorylated protein tyrosine; p-PLC γ-1: phosphorylated PLC γ-1; t-PLC γ-1: total PLC γ-1.

**Figure 5:**
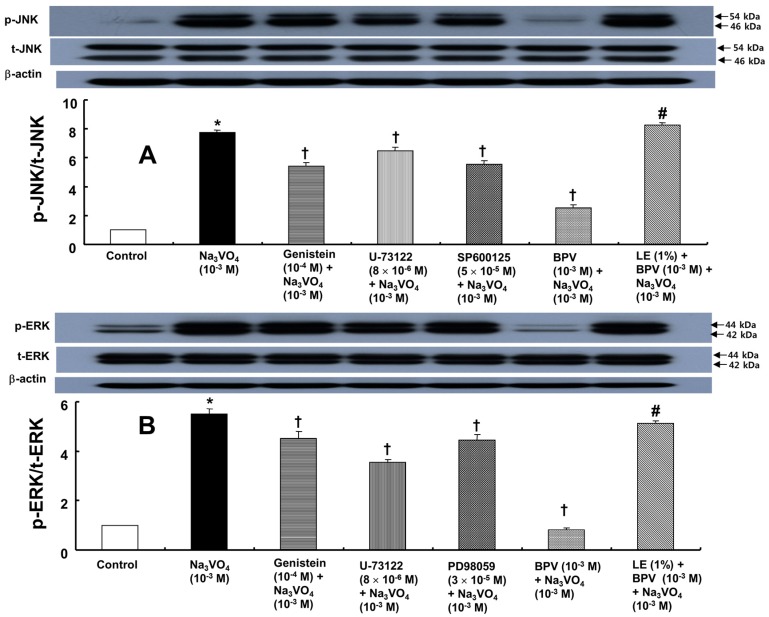
(**A**) Effects of genistein, U-73122, SP600125 and bupivacaine (BPV) alone and combined treatment with lipid emulsion (LE, 1%) and BPV on c-Jun NH_2_-terminal kinase (JNK) phosphorylation induced by sodium orthovanadate (Na_3_VO_4_, 10^−3^ M) in rat aortic vascular smooth muscle cells (RAVSMCs). RAVSMCs were treated with Na_3_VO_4_ alone for 30 min, pretreated with each inhibitor (genistein, U-73122 or SP600125) or BPV for 1 h and then treated with Na_3_VO_4_ for 30 min or pretreated with LE (1%) for 1 h followed by treatment with BPV for 1 h and then Na_3_VO_4_ for 30 min. JNK phosphorylation on Thr183/Tyr185 was examined by Western blot analysis as described in the Materials and Methods. The data are expressed as the means ± SD (*N* = 3). The effects of several inhibitors, BPV and combined treatment with LE and BPV on Na_3_VO_4_-induced JNK phosphorylation were analyzed using a one-way analysis of variance followed by Bonferroni’s post hoc test. *N* indicates the number of independent experiments. * *p* < 0.001 versus control. † *p* < 0.001 versus Na_3_VO_4_. # *p* < 0.001 versus combined treatment with BPV and Na_3_VO_4_. p-JNK: phosphorylated JNK; t-JNK: total JNK; (**B**) Effect of genistein, U-73122, PD98059 and BPV alone and combined treatment with LE (1%) and BPV on extracellular signal-regulated kinase (ERK) phosphorylation induced by Na_3_VO_4_ (10^−3^ M) in RAVSMCs. RAVSMCs were treated with Na_3_VO_4_ alone for 30 min, pretreated with either inhibitor (genistein, U-73122 or PD98059) or BPV for 1 h and then treated with Na_3_VO_4_ for 30 min or pretreated with LE (1%) for 1 h followed by treatment with BPV for 1 h and then Na_3_VO_4_ for 30 min. ERK phosphorylation on Thr202/Tyr204 was examined by Western blot analysis as described in the Materials and Methods. The data are expressed as the means ± SD (*N* = 3). The effects of several inhibitors, BPV and combined treatment with LE and BPV on Na_3_VO_4_-induced ERK phosphorylation were analyzed using a one-way analysis of variance followed by Bonferroni’s post hoc test. *N* indicates the number of independent experiments. * *p* < 0.001 versus control. † *p* < 0.001 versus Na_3_VO_4_. # *p* < 0.001 versus combined treatment with BPV and Na_3_VO_4_. p-ERK: phosphorylated ERK; t-ERK: total ERK.

**Figure 6:**
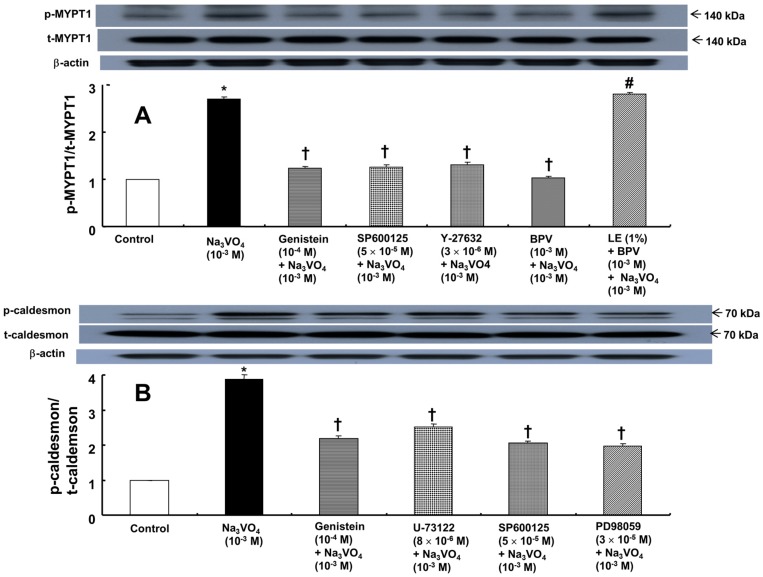
(**A**) Effects of genistein, SP600125, Y-27632 and bupivacaine (BPV) alone and combined treatment with lipid emulsion (LE, 1%) and BPV on myosin phosphatase target subunit 1 (MYPT1) phosphorylation induced by sodium orthovanadate (Na_3_VO_4_, 10^−3^ M) in rat aortic vascular smooth muscle cells (RAVSMCs). RAVSMCs were treated with Na_3_VO_4_ alone for 10 min, pretreated with each inhibitor (genistein, SP600125 or Y-27632) or BPV for 1 h and then treated with Na_3_VO_4_ for 10 min or pretreated with LE (1%) for 1 h followed by treatment with BPV for 1 h and then Na_3_VO_4_ for 10 min. MYPT1 phosphorylation on Thr696 was examined by Western blot analysis as described in the Materials and Methods. The data are expressed as the means ± SD (*N* = 3). The effects of several inhibitors, BPV and combined treatment with LE and BPV on Na_3_VO_4_-induced MYPT1 and caldesmon phosphorylation were analyzed using a one-way analysis of variance followed by Bonferroni’s post hoc test. *N* indicates the number of independent experiments. * *p* < 0.001 versus control. † *p* < 0.001 versus Na_3_VO_4_. # *p* < 0.001 versus combined treatment with BPV and Na_3_VO_4_. p-MYPT1: phosphorylated MYPT1; t-MYPT1: total MYPT1; (**B**) Effect of genistein, U-731225, SP600125 and PD98059 on caldesmon phosphorylation induced by Na_3_VO_4_ (10^−3^ M) in RAVSMCs. RAVSMCs were treated with Na_3_VO_4_ alone for 20 min, pretreated with each inhibitor (genistein, U-731225, SP600125 and PD98059) for 1 h and then treated with Na_3_VO_4_ for 20 min. Caldesmon phosphorylation on Ser789 was examined by Western blot analysis as described in the Materials and Methods. The data are expressed as the means ± SD (*N* = 3). *N* indicates the number of independent experiments. * *p* < 0.001 versus control. † *p* < 0.001 versus Na_3_VO_4_. p-caldesmon: phosphorylated caldesmon; t-caldesmon: total caldesmon.

Sodium orthovanadate (10^−3^ M) induced protein tyrosine and PLC γ-1 phosphorylation in rat aortic vascular smooth muscle cells (RAVSMCs) (Figure 4; *p* < 0.001 versus control). Genistein (10^−4^ M) and bupivacaine (10^−3^ M) attenuated the protein tyrosine and PLC γ-1 phosphorylation induced by sodium orthovanadate (Figure 4; *p* < 0.001 versus sodium orthovanadate alone), whereas the lipid emulsion (0.5% to 2%) attenuated the bupivacaine-mediated inhibition of sodium orthovanadate-induced protein tyrosine and PLC γ-1 phosphorylation (Figure 4; *p* < 0.001 versus bupivacaine plus sodium orthovanadate). Sodium orthovanadate (10^−3^ M) also induced JNK phosphorylation (Figure 5A; *p* < 0.001 versus control). Genistein (10^−4^ M), U-73122 (8 × 10^−6^ M), SP600125 (5 × 10^−5^ M) and bupivacaine (10^−3^ M) attenuated the induction of JNK phosphorylation by sodium orthovanadate (Figure 5A; *p* < 0.001 versus sodium orthovanadate alone), whereas the lipid emulsion (1%) attenuated the bupivacaine-mediated inhibition of sodium orthovanadate-induced JNK phosphorylation (Figure 5A; *p* < 0.001 versus bupivacaine plus sodium orthovanadate). Moreover, sodium orthovanadate (10^−3^ M) induced ERK phosphorylation (Figure 5B; *p* < 0.001 versus control). Genistein (10^−4^ M), U-73122 (8 × 10^−6^ M), PD98059 (3 × 10^−5^ M) and bupivacaine (10^−3^ M) attenuated the ERK phosphorylation induced by sodium orthovanadate (Figure 5B; *p* < 0.001 versus sodium orthovanadate alone), whereas the lipid emulsion (1%) attenuated the bupivacaine-mediated inhibition of sodium orthovanadate-induced ERK phosphorylation (Figure 5B; *p* < 0.001 versus bupivacaine plus sodium orthovanadate). In addition, sodium orthovanadate (10^−3^ M) induced MYPT1 phosphorylation (Figure 6A; *p* < 0.001 versus control). Genistein (10^−4^ M), SP600125 (5 × 10^−5^ M), Y-27632 (3 × 10^−6^ M) and bupivacaine (10^−3^ M) attenuated the MYPT1 phosphorylation induced by sodium orthovanadate (Figure 6A; *p* < 0.001 versus sodium orthovanadate alone), whereas the lipid emulsion (1%) attenuated the bupivacaine-mediated inhibition of sodium orthovanadate-induced MYPT1 phosphorylation (Figure 6A; *p* < 0.001 versus bupivacaine plus sodium orthovanadate). Finally, sodium orthovanadate (10^−3^ M) induced caldesmon phosphorylation (Figure 6B; *p* < 0.001 versus control), whereas genistein (10^−4^ M), U-731225 (8 × 10^−6^ M), SP600125 (5 × 10^−5^ M) and PD98059 (3 × 10^−5^ M) attenuated the caldesmon phosphorylation induced by sodium orthovanadate (Figure 6B; *p* < 0.001 versus sodium orthovanadate alone).

**Figure 7:**
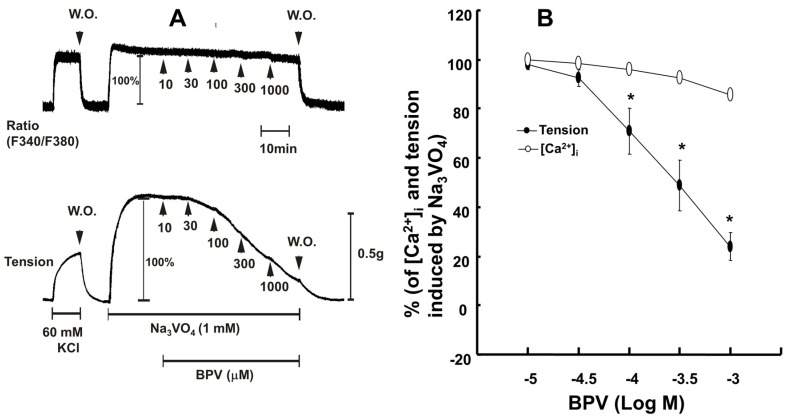
(**A**) Effects of bupivacaine (BPV) on the intracellular calcium level ([Ca^2+^]_i_) (upper trace) and tension (lower tracer) induced by sodium orthovanadate (Na_3_VO_4_, 10^−3^ M) in isolated endothelium-denuded rat aortic strips. The [Ca^2+^]_i_ of fura-2-loaded aortic strips was determined using a fluorometer and is presented as the F340/F380 ratio. A value of 100% represents the Na_3_VO_4_ (10^−3^ M)-induced increase in both [Ca^2+^]_i_ and tension before the cumulative addition of BPV. When the [Ca^2+^]_i_ and tension evoked by Na_3_VO_4_ reached a plateau, BPV (10^−5^ to 10^−3^ M) was cumulatively added to the organ bath. W.O.: washout; (**B**) BPV concentration-response curves in aortic strips precontracted with Na_3_VO_4_ (10^−3^ M). BPV was added during sustained [Ca^2+^]_i_ and tension induced by Na_3_VO_4_. The value of 100% indicates sustained [Ca^2+^]_i_ and tension induced by Na_3_VO_4_ before the addition of BPV. The data represent the means ± SD (*N* = 5). The effects of BPV on [Ca^2+^]_i_ and tension induced by Na_3_VO_4_ were analyzed using a two-way repeated-measures analysis of variance (ANOVA) or repeated-measures ANOVA followed by Bonferroni’s post hoc test. *N* indicates the number of independent experiments. * *p* < 0.001 versus [Ca^2+^]_i_.

Bupivacaine (10^−4^ to 10^−3^ M) inhibited the contraction and intracellular calcium level ([Ca^2+^]_i_) induced by sodium orthovanadate (10^−3^ M) (*p* < 0.001 versus sodium orthovanadate alone). However, the magnitude of the bupivacaine-induced vasodilation was greater than that of the bupivacaine-induced [Ca^2+^]_i_ decrease during sodium orthovanadate-evoked contraction (Figure 7B; *p* < 0.001 at 10^−4^ to 10^−3^ M bupivacaine).

## 3. Discussion

This is the first study to suggest that lipid emulsions reverse toxic-dose bupivacaine-induced vasodilation via the activation of a pathway involving tyrosine kinase, JNK, MYPT1, PLC γ-1 and ERK during sodium orthovanadate-induced protein tyrosine phosphorylation-mediated contraction, and this phenomenon appears to be associated with the lipid solubility of the local anesthetics. The major findings of this in vitro study are as follows: (1) a lipid emulsion reversed the vasodilation induced by bupivacaine; (2) genistein, SP600125 and Y-27632 greatly attenuated sodium orthovanadate-induced contraction; (3) a lipid emulsion attenuated the bupivacaine-mediated inhibition of protein tyrosine, JNK, MYPT1, PLC γ-1 and ERK phosphorylation induced by sodium orthovanadate; and (4) bupivacaine-induced vasodilation was greater than the bupivacaine-induced reduction in [Ca^2+^]_i_.

Genistein and Y-27632 have been reported to inhibit the contraction induced by sodium orthovanadate [6,7,8,9]. Similar to previous reports, genistein, Y-27632 and SP600125 highly attenuated sodium orthovanadate-induced contraction in the present study [6,7,8,9]. Furthermore, PD98059 has been previously shown to attenuate sodium orthovanadate-induced contraction in rat mesenteric arteries [9]. Consistently, in the current study, PD98059 and U-73122 inhibited the contraction evoked by sodium orthovanadate [9]. Taken together, these data indicate that the contraction induced by sodium orthovanadate appears to be primarily mediated by a pathway involving tyrosine kinase, JNK and Rho-kinase and partially by a pathway involving PLC γ-1 and ERK.

In agreement with previous reports, sodium orthovanadate induced protein tyrosine, PLC γ-1 and ERK phosphorylation [6,9]. In addition, the tyrosine kinase inhibitor genistein attenuated protein tyrosine and PLC γ-1 phosphorylation induced by sodium orthovanadate. Genistein, the PLC γ-1 inhibitor U-73122, the ERK inhibitor PD98059 or the JNK inhibitor SP600125 inhibited ERK or JNK phosphorylation induced by sodium orthovanadate. Taken together with the above results and the tension study, these findings indicate that sodium orthovanadate-induced contraction is mediated by JNK or ERK, which are activated by tyrosine kinase-induced PLC γ-1 phosphorylation. Vascular smooth muscle contraction is regulated by an increase in intracellular calcium and calcium sensitization [4,11]. Calcium sensitization is mediated by the inhibition of myosin light chain phosphatase, which leads to the increased phosphorylation of the 20-kDa regulatory light chain of myosin and subsequently enhanced contraction [4,11]. The inhibition of myosin light chain phosphatase is induced by MYPT1 phosphorylation activated by Rho-kinase [4,11]. In the current study, sodium orthovanadate induced MYPT1 phosphorylation, which was inhibited by genistein, SP600125 and Y-27632. Similar to previous reports and taken together with the tension study, these results suggest that sodium orthovanadate-induced MYPT1 phosphorylation is mediated by a pathway involving tyrosine kinase, JNK and Rho-kinase [8,9]. However, MYPT1 phosphorylation induced by sodium orthovanadate in rat mesenteric arteries is mediated by a pathway involving Rho-kinase activated by ERK [9]. The different MAPK isoform involved in MYPT1 phosphorylation induced by sodium orthovanadate may be due to differences in the vessel (aorta versus mesenteric artery) [9]. Previous studies have reported that a toxic dose of bupivacaine attenuates the phosphorylation of MYPT1 and the phosphorylation-dependent inhibitory protein of myosin phosphatase (CPI-17) induced by the Rho-kinase stimulant NaF and protein kinase C stimulant phorbol 12,13-dibutyrate (PDBu), respectively, leading to decreased calcium sensitization [12,13]. In addition, bupivacaine has been reported to inhibit phenylephrine-induced contraction via decreased calcium sensitization [14]. Taken together, as bupivacaine attenuated sodium orthovanadate-induced protein tyrosine and JNK phosphorylation in the current study, these findings indicate that decreased calcium sensitization as a result of bupivacaine-mediated inhibition of MYPT1 phosphorylation is due to the inhibition of a pathway involving tyrosine kinase and JNK [4,12,13,14]. As CPI-17 phosphorylation induced by protein kinase C and Rho-kinase contributes to calcium sensitization via the inhibition of myosin light chain phosphatase, further studies regarding the effect of sodium orthovanadate on CPI-17 phosphorylation are needed to elucidate the detailed cellular signaling pathways [11]. As sodium orthovanadate-induced contraction involves Rho-kinase-induced phosphorylation of MYPT1, which is located downstream of the phosphorylation of Src and epidermal growth factor receptor, further studies are needed to examine the effect of bupivacaine on the phosphorylation of Src and epidermal growth factor receptor induced by sodium orthovanadate to elucidate the mechanism underlying bupivacaine-induced vasodilation [8,9]. In addition, as bupivacaine also inhibited the phosphorylation of protein tyrosine kinase, PLC γ-1 and ERK induced by sodium orthovanadate, bupivacaine-induced vasodilation appears to be mediated by the inhibition of another signaling pathway involving tyrosine kinase, PLC γ-1 and ERK. Thus, additional analyses are needed to identify the cellular signaling pathways downstream of tyrosine kinase, PLC γ-1 and ERK associated with sodium orthovanadate-induced contraction. The inhibitory actin-binding protein caldesmon attenuates the actin-myosin interaction, modulating vascular smooth muscle contraction [15]. Caldesmon phosphorylation mediated by tyrosine kinase-induced MAPK phosphorylation attenuates the inhibitory effect of caldesmon on the actin-myosin interaction, leading to increased contraction [4,15]. Genistein, U-73122, SP600125 and PD98059 inhibited sodium orthovanadate-induced caldesmon phosphorylation, which suggests that caldesmon phosphorylation is mediated by a pathway involving tyrosine kinase, PLC γ-1, JNK and ERK.

In agreement with previous reports, sodium orthovanadate induced MYPT1 phosphorylation, suggesting that sodium orthovanadate-induced contraction is mediated by calcium sensitization [8,9,11]. However, in the current study, a toxic dose of bupivacaine inhibited sodium orthovanadate-induced MYPT1 phosphorylation. It has been reported that bupivacaine inhibits calcium sensitization in aortae precontracted with PDBu, NaF and phenylephrine [12,13,14]. In agreement with previous reports and the present Western blot results, bupivacaine decreased more tension than [Ca^2+^]_i_ during sodium orthovanadate-induced contraction, suggesting that the bupivacaine-induced reduction in calcium sensitization is mediated by the inhibition of sodium orthovanadate-induced MYPT1 phosphorylation [12,13,14]. Thus, the lipid emulsion-mediated reversal of bupivacaine-evoked inhibition of MYPT1 phosphorylation induced by sodium orthovanadate may be associated with the reversal of decreased calcium sensitization.

**Figure 8:**
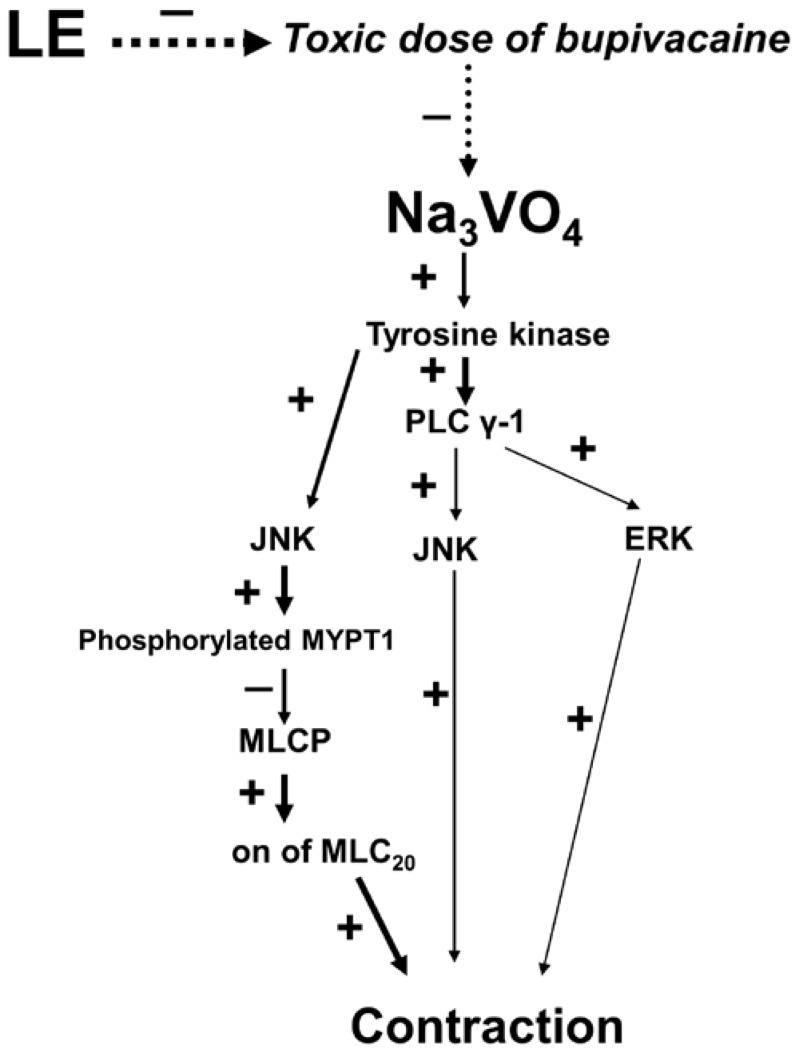
The putative cellular signaling pathways responsible for the lipid emulsion (LE)-mediated inhibition of toxic-dose bupivacaine-induced vasodilation during sodium orthovanadate (Na_3_VO_4_)-induced contraction in isolated endothelium-denuded rat aortae. PLC γ-1: phospholipase C γ-1, JNK: c-Jun NH_2_-terminal kinase, ERK: extracellular signal-regulated kinase, MYPT1: myosin phosphatase target subunit 1; MLCP: myosin light chain phosphatase; MLC_20_: 20-kDa regulatory light chain of myosin. +: stimulation; −: inhibition; Solid line: stimulation pathway; Dotted line: inhibitory pathway.

Bupivacaine, which is a racemic form, produces more potent cardiovascular toxicity than the S (−) isomer levobupivacaine [16]. It has been reported that lipid emulsions are effective for the treatment of systemic toxicity induced by local anesthetics, including bupivacaine, and non-local anesthetic drugs, including calcium channel blockers, tricyclic antidepressants and antipsychotics [1,17]. Taken together with the tension measurements and Western blot results, these results suggest that lipid emulsion reverses the inhibitory effect of bupivacaine on protein phosphorylation involving tyrosine kinase, JNK, MYPT1, PLC γ-1 and ERK activated by sodium orthovanadate (Figure 8). As lipid emulsion is used to treat systemic toxicity following bupivacaine-induced cardiovascular collapse, the order of drug treatment in the present tension study was sodium orthovanadate, bupivacaine and lipid emulsion. However, the order of drug treatment used in the present Western blot study was lipid emulsion, bupivacaine and sodium orthovanadate because the cellular signaling pathway associated with the lipid emulsion-mediated attenuation of the bupivacaine-induced inhibition of protein phosphorylation evoked by sodium orthovanadate was investigated. The underlying mechanisms associated with lipid emulsion treatment include a lipid sink, increased fatty acid supply, glycogen synthase kinase-3β phosphorylation-induced inhibition of mitochondria permeability transition pore opening and lipid shuttle [1]. The lipid sink theory, which is widely accepted among the underlying mechanisms, indicates that lipid emulsions sequester large amounts of highly lipid-soluble drugs compared with less lipid-soluble drugs [1]. Lipid emulsions attenuate the vasodilation induced by a toxic dose of the highly lipid-soluble local anesthetic bupivacaine in isolated endothelium-denuded rat aortae precontracted with NaF or PDBu, whereas lipid emulsions have no effect on the vasodilation induced by a toxic dose of the less-lipid-soluble local anesthetic mepivacaine [12,13]. All of these previous reports suggest that the magnitude of the lipid emulsion-mediated attenuation and reversal of vasodilation induced by a toxic dose of a local anesthetic appears to be correlated with the lipid solubility of local anesthetics [2,3,12,13]. In addition, pretreatment or posttreatment with a lipid emulsion has been reported to be effective for recovery from toxic-dose bupivacaine-induced cardiac arrest, but it has no effect on toxic-dose mepivacaine-induced cardiac arrest [18,19]. Similar to previous reports, the decreasing order of magnitude of lipid emulsion-mediated reversal of toxic-dose local anesthetic-induced vasodilation during sodium orthovanadate-induced protein tyrosine phosphorylation-mediated contraction in the current study was as follows: bupivacaine, lidocaine and mepivacaine (Supplementary Figure S1) [2,3,12,13,18,19]. As high concentrations of lipid emulsion (1.75% and 2%) slightly reduced the contraction induced by sodium orthovanadate rather than increasing it, the magnitude of the lipid emulsion-mediated reversal of toxic-dose local anesthetic-induced vasodilation in the current study appears to be correlated with the lipid solubility of local anesthetics (lipid solubility [lipid/H_2_O]: bupivacaine [27.5], lidocaine [2.9], and mepivacaine [0.8]) [20]. However, lipid emulsions have been shown to attenuate the decreased sodium current induced by the less lipid-soluble mepivacaine in cardiomyocytes, and long chain fatty acids activate voltage-dependent calcium channels in ventricular myocytes [21,22]. A high dose of lipid emulsion also slightly reversed mepivacaine-induced vasodilation in the present study; thus, further analyses are needed to determine the effect of the lipid emulsion on the local anesthetic-induced changes in calcium channel currents and intracellular calcium concentration in RAVSMCs. A lipid emulsion attenuated bupivacaine-induced cardiotoxicity via opioid receptors in vivo [23]. However, the current results suggest that opioid receptors do not contribute to the lipid emulsion-mediated reversal of toxic-dose bupivacaine-induced vasodilation. This difference may be ascribed to differences in the organ (aorta versus heart) and experimental methods (in vitro versus in vivo) [23]. The current in vitro study has several limitations. First, we studied the aorta, which is regarded as a conduit vessel, whereas small resistance arterioles mainly contribute to blood pressure by regulating systemic vascular resistance [24]. Second, lipid emulsion inhibits acetylcholine-induced nitric oxide-mediated relaxation in isolated endothelium-intact aortae [25]. In addition, the vasoconstriction induced by local anesthetics, including levobupivacaine, mepivacaine and ropivacaine, has been reported to be attenuated by endothelial nitric oxide [26,27,28]. Furthermore, it has been reported that the lipid emulsion-mediated inhibition of levobupivacaine-induced endothelial nitric oxide synthase phosphorylation partially contributes to the lipid emulsion-mediated reversal of toxic-dose levobupivacaine-induced vasodilation [3,29]. Endothelial nitric oxide has been reported to attenuate sodium orthovanadate-induced contraction [9]. Taking into consideration previous reports, we hypothesized that the magnitude of the lipid emulsion-mediated reversal of toxic-dose bupivacaine-induced vasodilation during sodium orthovanadate-induced contraction may be greatly enhanced in endothelium-intact aortae compared with endothelium-denuded aortae [3,9,25,26,27,28,29,30,31]. However, we used l-NAME-pretreated endothelium-denuded rat aortae to focus on the vascular smooth muscle and to avoid confounding factors, such as vasodilation induced by endothelial nitric oxide released by local anesthetics and sodium orthovanadate [9,26,27,28,30,31]. Third, vascular tone is regulated by extrinsic factors in vivo, including the sympathetic nervous system and circulating hormones [4]. These factors may have modified the lipid emulsion-mediated reversal of toxic-dose bupivacaine-induced vasodilation observed in the current study. However, even with these limitations, lipid emulsion treatment appeared to provide beneficial effects in terms of vascular tone recovery from vascular collapse induced by a toxic concentration (10^−3^ M) of bupivacaine exceeding the serum concentration (10^−5^ M), which causes systemic toxicity [32].

## 4. Materials and Methods

All experimental procedures and protocols (GNU-160414-R0019; 6 April 2016) were approved by the Institutional Animal Care and Use Committee at Gyeongsang National University and Catholic Kwandong University. All experimental procedures were performed according to the Guide for the Care and Use of Laboratory Animals prepared by the Institute for Laboratory Animal Research.

### 4.1. Preparation of Aortic Rings for Tension Measurements

Aortic rings were isolated and prepared for tension measurements as previously described [12,33]. Male Sprague-Dawley rats weighing 250–300 g were fully anesthetized by inhalation of 100% carbon dioxide. The descending thoracic aorta was removed and dissected from the connective tissue and fat surrounding the aorta under microscopic guidance in Krebs solution. The aorta was then cut to a length of 2.5 mm and suspended in Grass isometric transducers (FT-03, Grass Instrument, Quincy, MA, USA) under a 3.0-g resting tension in a 10-mL Krebs organ bath at 37 °C. The aorta was continuously aerated using 95% O_2_ and 5% CO_2_ to maintain pH values from 7.35 to 7.45. A 3.0-g resting tension was used to equilibrate the rings for 120 min, and the bath solution was changed every 30 min. A 25-gauge needle was inserted into the aortic lumen, and all the aortic rings were rubbed for several seconds to remove the endothelium. To confirm the endothelial denudation, we added phenylephrine (10^−8^ M) to the organ bath with endothelium-denuded aortic rings, and the phenylephrine produced a sustained and stable contraction. Next, we added acetylcholine (10^−5^ M) to the organ bath containing aortic rings with phenylephrine-induced contraction, and endothelial denudation was confirmed by observing less than 15% acetylcholine-induced relaxation from phenylephrine-induced contraction. After returning the resting tension to baseline by exchanging the Krebs solution containing phenylephrine with fresh Krebs solution, the following experiments were performed. A single ring was used to obtain dose-response curves induced by lipid emulsion (Intralipid^®^, Fresenius Kabi Korea, Seoul, Korea) or various inhibitors. Sodium orthovanadate-induced contraction is attenuated by endothelial nitric oxide [9,30]. Thus, to avoid the effect of residual endothelium on sodium orthovanadate-induced contraction in this experiment, the following experimental protocols were used for all endothelium-denuded aortae that were pretreated with N^w^-nitro-l-arginine methyl ester (l-NAME, 10^−4^ M).

### 4.2. Experimental Protocols

First, we investigated the effect of a lipid emulsion on vasodilation induced by a toxic dose of local anesthetics in l-NAME-pretreated endothelium-denuded rat aortae precontracted with sodium orthovanadate. In addition, some l-NAME-pretreated endothelium-denuded aortae were pretreated with naloxone (10^−5^ M) for 15 min before the addition of sodium orthovanadate to investigate whether the lipid emulsion-mediated reversal of bupivacaine-induced vasodilation involved the activation of opioid receptors [23]. After sodium orthovanadate (10^−3^ M) produced a sustained and stable contraction in the presence or absence of naloxone, a toxic dose of local anesthetic (10^−3^ M bupivacaine, 3 × 10^−3^ M lidocaine and 10^−2^ M mepivacaine) was added to the organ bath to induce vasodilation. Subsequently, a lipid emulsion (0.25% to 2%) was cumulatively added to obtain lipid emulsion dose-response curves in the vasodilated aortae induced by a toxic dose of local anesthetic. In addition, we investigated the effect of lipid emulsion alone on the sodium orthovanadate-induced contraction or resting tension of isolated endothelium-denuded rat aortae. After sodium orthovanadate (10^−3^ M) produced a sustained and stable contraction in isolated endothelium-denuded rat aortae pretreated with l-NAME (10^−4^ M), lipid emulsion (0.25% to 2%) was cumulatively added to the sodium orthovanadate-induced contracted aortae. Lipid emulsion was also cumulatively added to l-NAME (10^−4^ M)-pretreated endothelium-denuded rat aortae without sodium orthovanadate.

Second, we examined the effects of genistein, U-73122, SP600125, PD98059 and Y-27632 on contraction induced by sodium orthovanadate (10^−3^ M) in l-NAME-pretreated isolated endothelium-denuded rat aortae. After sodium orthovanadate (10^−3^ M) produced a sustained and stable contraction, genistein (3 × 10^−6^ to 10^−4^ M), U-73122 (10^−6^ to 10^−4^ M), SP600125 (3 × 10^−6^ to 3 × 10^−5^ M), PD98059 (10^−5^ to 10^−4^ M) and Y-27632 (10^−7^ to 3 × 10^−6^ M) were cumulatively added to the vasoconstricted aortae induced by orthovanadate (10^−3^ M).

### 4.3. Cell Culture

RAVSMCs were isolated from rat thoracic aortae by enzymatic dissociation and then cultured in Dulbecco’s modified Eagle’s medium (HyClone, GE Healthcare, Salt Lake City, UT, USA) supplemented with 10% heat-inactivated fetal bovine serum (Gibco, Life Technologies, New York, NY, USA), 2 mM l-glutamine, 100 U/mL penicillin and 100 µg/mL streptomycin as previously described [33]. The cells were plated onto a 100-mm culture dish and incubated at 37 °C in a humidified atmosphere containing 5% CO_2_, and the medium was changed every alternate day until the cells reached confluence. Upon confluence, the cells were dissociated with 0.05% trypsin-ethylenediaminetetraacetic acid solution and replated at a 1:4 ratio. For this study, cells between passages 2 and 10 were seeded (10^7^ cells/100 mm dish) and cultured until they reached 70% confluence, followed by serum starvation overnight prior to drug treatment.

### 4.4. Western Blot Analysis

Western blot analysis was performed per a previously reported protocol [33]. Total protein concentrations were determined by employing the Bradford method [34]. Protein samples from cell lysates were mixed with an equal volume of 2× sodium dodecyl sulfate sample buffer (0.1 M Tris-HCl, 20% glycerol, 4% sodium dodecyl sulfate and 0.01% bromophenol blue). Aliquots (30 µg) of proteins were separated by 7% or 10% sodium dodecyl sulfate-polyacrylamide gel electrophoresis for 90 min at 110 V. The separated proteins were electrophoretically transferred to polyvinylidene difluoride membranes at 190 mA for 1 h. The membranes were then blocked with 5% *w*/*v* nonfat dried milk in Tris-buffered saline containing Tween-20 (TBST) for 2 h at room temperature, followed by incubation with specific primary antibodies (anti-phospho-tyrosine, anti-phospho-PLC γ-1, anti-phospho-JNK, anti-phospho-ERK, anti-phospho-MYPT, anti-PLC γ-1, anti-JNK, anti-ERK, anti-MYPT, anti-phospho-caldesmon, anti-caldesmon and anti-β-actin) diluted 1:1000 in TBST containing 5% *w*/*v* skim milk at 4 °C overnight. After incubation, the membranes were washed 3 times with TBST and incubated with secondary antibodies tagged with horseradish peroxidase-conjugated anti-rabbit or anti-mouse IgG diluted 1:5000 in TBST containing 5% *w*/*v* skim milk for 1 h at room temperature. The membranes were washed in TBST, and the immunoreactive signals were detected using enhanced chemiluminescence (SuperSignal^®^ West Pico Chemiluminescent Substrate; Thermo Scientific, Rockford, IL, USA) and transferred onto an X-ray film (^Super^RX-N Fuji Medical X-ray Film, Tokyo, Japan). The band intensity was measured using densitometry.

### 4.5. Fura-2 Loading and Simultaneous Measurement of [Ca^2+^]_i_ and Tension

The loading of fura-2 and the simultaneous measurement of [Ca^2+^]_i_ and tension were performed as previously reported by our laboratory [33]. Male Sprague-Dawley rats (250–300 g, *N* = 20) were sacrificed by intraperitoneal injection of sodium pentobarbital (50 mg/mL) followed by exsanguination. The descending thoracic aorta was isolated and dissected free from the surrounding connective tissue and fat under microscopic guidance and was placed in Krebs solution (118 mM NaCl, 4.7 mM KCl, 1.2 mM MgSO_4_, 1.2 mM KH_2_PO_4_, 2.5 mM CaCl_2_, 25 mM NaHCO_3_ and 11 mM glucose). [Ca^2+^]_i_ was measured using the fluorescent Ca^2+^ indicator fura-2. Muscle strips were treated with acetoxymethyl ester of fura-2 (fura-2/AM, 10 µM) in the presence of 0.02% Cremophor EL for 5–6 h at room temperature. Fura-2-loaded muscle strips were then transferred to a temperature-controlled 7-mL organ bath in a fluorometer (CAF-100; Jasco, Tokyo, Japan) and rinsed with fresh Krebs solution at 37 °C for 20 min to remove unhydrolyzed fura-2/AM. Isometric contraction of the smooth muscle was recorded using a force-displacement transducer (MLT050, ADInstruments, Colorado Springs, CO, USA). The muscle strips were illuminated alternately (48 Hz) with excitation wavelengths of 340 and 380 nm. The light emitted from the muscle strips (F340 and F380) was collected by a photomultiplier through a 500-nm filter, and the ratio of F340/F380 was used as a measure of [Ca^2+^]_i_. The absolute [Ca^2+^]_i_ was not calculated in the current experiment because the dissociation constant of the fluorescence indicator for Ca^2+^ in the cytosol may differ from that measured under in vitro conditions [35]. Therefore, the F340/F380 ratios and contraction caused by sodium orthovanadate (10^−3^ M) were considered to be 100% of the [Ca^2+^]_i_ and contraction, respectively. Muscle tension and F340/F380 ratios were recorded with PowerLab/400 using the Chart program (ADInstruments). Muscle strips were placed under an initial 3.0-g resting tension. When both the [Ca^2+^]_i_ and contraction evoked by sodium orthovanadate (10^−3^ M) reached a sustained level, bupivacaine (10^−5^ to 10^−3^ M) was cumulatively added to the organ bath.

### 4.6. Materials

All drugs used in this experiment were the highest commercially available purity. Bupivacaine and lidocaine were purchased from Reyon Pharmaceutical Co., Ltd. (Seoul, Korea). Mepivacaine was obtained from Hana Pharmaceutical Co., Ltd. (Gyeonggi-do, Korea). Sodium orthovanadate, genistein, U-73122, SP600125, PD98059 and anti-β-actin antibody were obtained from Sigma-Aldrich (St. Louis, MO, USA). Y-27632 was obtained from Calbiochem (La Jolla, CA, USA). Anti-phospho-tyrosine, anti-phospho-PLC γ-1 (Tyr783), anti-phospho-JNK (Thr183/Tyr185), anti-phospho-ERK (Thr202/Tyr204), anti-phospho-MYPT (Thr696), anti-PLC γ-1, anti-JNK, anti-ERK and anti-MYPT antibodies were obtained from Cell Signaling Technology (Beverly, MA, USA). Anti-phospho-caldesmon (Ser789) and anti-caldesmon antibodies were obtained from Millipore (Billerica, MA, USA) and Abcam (Cambridge Science Park, Cambridge, England), respectively. Fura-2/AM was purchased from Molecular Probes (Eugene, OR, USA). All concentrations are expressed as the final molar concentration. Genistein, U-73122, SP600125 and PD98059 were dissolved in DMSO. Unless otherwise stated, all drugs were dissolved and diluted in distilled water.

### 4.7. Data Analysis

The values are shown as the means ± SD. Vascular responses (vasodilation or vasoconstriction) induced by local anesthetics (bupivacaine, lidocaine and mepivacaine), lipid emulsion and various inhibitors (genistein, U-73122, SP600125, PD98059 and Y-27632) during sodium orthovanadate-induced contraction are expressed as the percentage of maximal sodium orthovanadate-induced contraction. The simultaneously measured values of [Ca^2+^]_i_ and tension induced by bupivacaine are expressed as the percentage of [Ca^2+^]_i_ and tension induced by sodium orthovanadate, respectively. For these data, a normality test was performed. As effect size and previous similar experiments were not available, we used the resource equation method to calculate the sample size in each group [36]. According to the resource equation method, the sample size in two and three groups should be from 6 to 11 and from 5 to 8, respectively [36]. *p*-values less than 0.05 were considered statistically significant.

## 5. Conclusions

In conclusion, lipid emulsion reversed toxic-dose bupivacaine-induced vasodilation via the activation of a pathway involving either tyrosine kinase, JNK, Rho-kinase and MYPT1 or tyrosine kinase, PLC γ-1 and ERK during sodium orthovanadate-induced protein tyrosine phosphorylation-mediated contraction (Figure 8). This reversal appears to be associated with the lipid solubility of the local anesthetic and partially due to the recovery of vascular smooth muscle cells from decreased calcium sensitization.

## Supplementary Materials

Supplementary materials can be found at www.mdpi.com/1422-0067/18/2/394/s1.

## Abbreviations

JNK	c-Jun NH_2_-terminal kinase
MYPT1	Myosin phosphatase target subunit 1
PLC γ-1	Phospholipase C γ-1
ERK	Extracellular signal-regulated kinase
[Ca^2+^]_i_	Intracellular calcium level
RAVSMCs	Rat aortic vascular smooth muscle cells
CPI-17	Phosphorylation-dependent inhibitory protein of myosin phosphatase
PDBu	Phorbol 12,13-dibutyrate
