# Supplementary material for: A Lipid Emulsion Reverses Toxic-Dose Bupivacaine-Induced Vasodilation during Tyrosine Phosphorylation-Evoked Contraction in Isolated Rat Aortae
Source: Int J Mol Sci. 2017 Feb 13;18(2):394. doi: 10.3390/ijms18020394 (PMC5343929; doi:10.3390/ijms18020394)
Supplement: Supplementary file 1 [file ijms-18-00394-s001.pdf]

Supplementary Materials: A Lipid Emulsion Reverses Toxic-Dose Bupivacaine-Induced Vasodilation during Tyrosine Phosphorylation-Evoked Contraction in Isolated Rat Aortae

Seong-Ho Ok, Soo Hee Lee, Seong-Chun Kwon, Mun Hwan Choi, Il-Woo Shin, Sebin Kang, Miyeong Park, Jeong-Min Hong and Ju-Tae Sohn

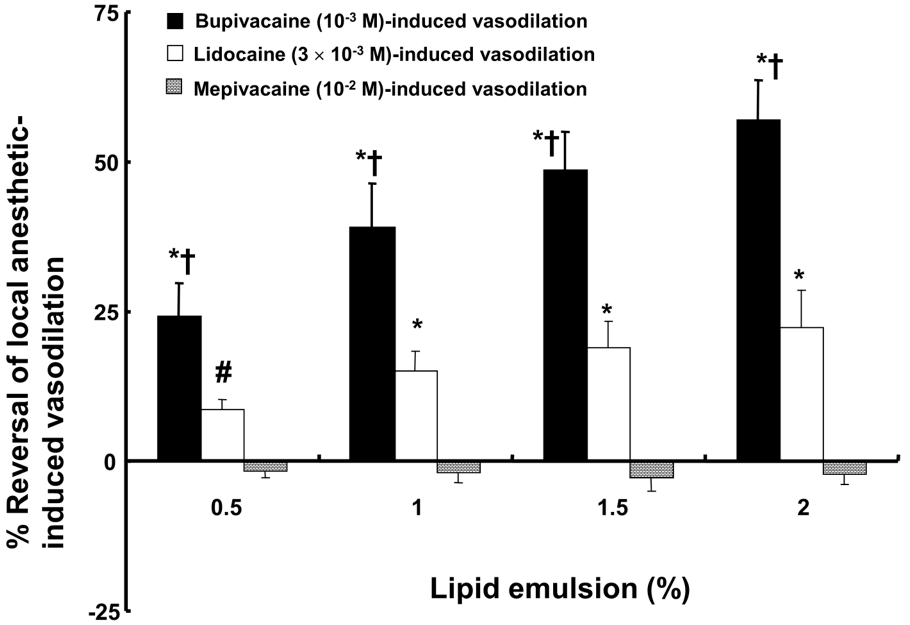

Figure S1. The magnitude of the lipid emulsion-mediated reversal of vasodilation induced by bupivacaine (10^{-3} M, $N = 5$), lidocaine (3×10^{-3} M, $N = 5$) and mepivacaine (10^{-2} M, $N = 5$) during sodium orthovanadate (10^{-3} M)-induced contraction in isolated endothelium-denuded rat aortae. The data represent the percentage of the reversal of local anesthetic-induced vasodilation from sodium orthovanadate-induced contraction. The magnitude of the lipid emulsion-mediated reversal of local anesthetic-induced vasodilation was analyzed using a two-way repeated-measures analysis of variance followed by Bonferroni's post hoc test. *N* indicates the number of rats from which descending thoracic aortic rings were derived. # $p < 0.05$ and * $p < 0.001$ versus mepivacaine (10^{-2} M)-induced vasodilation. † $p < 0.001$ versus lidocaine (3×10^{-3} M)-induced vasodilation.
